# Comparison of the Gene Coding Contents and Other Unusual Features of the GC-Rich and AT-Rich Branch Probosciviruses

**DOI:** 10.1128/mSphere.00091-16

**Published:** 2016-06-15

**Authors:** Paul D. Ling, Simon Y. Long, Jian-Chao Zong, Sarah Y. Heaggans, Xiang Qin, Gary S. Hayward

**Affiliations:** a Baylor College of Medicine, Houston, Texas, USA; b Viral Oncology Program, The Johns Hopkins School of Medicine, Baltimore, Maryland, USA; c The Human Genome Sequencing Center, Houston, Texas, USA; UNC-Chapel Hill

**Keywords:** elephant herpesviruses, acute hemorrhagic disease, evolutionary divergence, proposed *Deltaherpesvirinae* subfamily

## Abstract

Nearly 100 cases of lethal acute hemorrhagic disease in young Asian elephants have been reported worldwide. All tested cases contained high levels of elephant endotheliotropic herpesvirus (EEHV) DNA in pathological blood or tissue samples. Seven known major types of EEHVs have been partially characterized and shown to all belong to the novel *Proboscivirus* genus. However, the recently determined 206-kb EEHV4 genome proved to represent the prototype of a GC-rich branch virus that is very distinct from the previously published 180-kb EEHV1A, EEHV1B, and EEHV5A genomes, which all fall within an alternative AT-rich branch. Although EEHV4 retains the large family of 7xTM and vGPCR-like genes, six are unique to either just one or the other branch. While both branches display a highly enriched distribution of A and T tracts in intergenic domains, they are generally much larger within the GC-rich branch. Both branches retain the vGCNT1 acetylglucosamine transferase and at least one vOX-2 gene, but the two branches differ by 25 genes overall, with the AT-rich branch encoding a fucosyl transferase (vFUT9) plus two or three more vOX2 proteins and an immunoglobulin-like gene family that are all absent from the GC-rich branch. Several envelope glycoproteins retain only 15 to 20% protein identity or less across the two branches. Finally, the two plausible predicted transcriptional regulatory proteins display no homology at all to those in the alpha-, beta-, or gammaherpesvirus subfamilies. These results reinforce our previous proposal that the probosciviruses should be designated a new subfamily of mammalian herpesviruses.

**IMPORTANCE** Multiple species of herpesviruses from three different lineages of the *Proboscivirus* genus (EEHV1/6, EEHV2/5, and EEHV3/4/7) infect either Asian or African elephants, but the highly lethal hemorrhagic disease is largely confined to Asian elephant calves and is predominantly associated with EEHV1. In the accompanying paper [P. D. Ling et al., mSphere 1(3):e00081-15, 10.1128/mSphere.00081-15], we report the complete 206-kb genome of EEHV4, the third different species causing disease in Asian elephants and the first example of a GC-rich branch proboscivirus. To gain insights into the nature and differential properties of these two very anciently diverged lineages of elephant herpesviruses, we describe here several additional unusual features found in the complete GC-rich genome of EEHV4 with particular emphasis on patterns of divergence as well as common unique features that are distinct from those of all other herpesviruses, such as the enlarged AT-rich intergenic domains and gene families, including the large number of vGPCR-like proteins.

## Abstract (precis)

Multiple species of herpesviruses from three different lineages of the *Proboscivirus* genus (EEHV1/6, EEHV2/5, and EEHV3/4/7) infect either Asian or African elephants, but the highly lethal hemorrhagic disease is largely confined to Asian elephant calves and is predominantly associated with EEHV1. In the accompanying paper [P. D. Ling et al., mSphere 1(3):e00081-15, http://dx.doi.org/10.1128/mSphere.00081-15], we report the complete 206-kb genome of EEHV4, the third different species causing disease in Asian elephants and the first example of a GC-rich branch proboscivirus. To gain insights into the nature and differential properties of these two very anciently diverged lineages of elephant herpesviruses, we describe here several additional unusual features found in the complete GC-rich genome of EEHV4 with particular emphasis on patterns of divergence as well as common unique features that are distinct from those of all other herpesviruses, such as the enlarged AT-rich intergenic domains and gene families, including the large number of vGPCR-like proteins.

## INTRODUCTION

In addition to all of the other factors threatening the survival and breeding success of endangered Asian elephants, about 20% of juvenile Asian elephants in North America and Europe, and up to 43 calves in Asian range countries as well, are known to have died from an acute hemorrhagic disease associated with elephant endotheliotropic herpesvirus (EEHV) (1–6). Most lethal cases as well as several survivors have been confirmed by DNA PCR analyses to have high-level systemic infections by one of seven distinct species of elephant herpesviruses named EEHV1 to EEHV7 that form a single clade within the *Proboscivirus* genus (1, 2, 7–10). Although EEHV1 has been most commonly identified in over 90% of lethal cases, EEHV4 and EEHV5 have also been found in several lethal and nonlethal examples of hemorrhagic disease within Asian elephants (*Elephas maximus*) (11–15). Just three additional viremic disease cases that were associated with either EEHV2, EEHV3, or EEHV6 have been detected in African elephant calves (*Loxodonta africana*) (7–9, 16).

Close monitoring of blood, trunk washes, and saliva from apparently healthy adults in several zoo herds has also demonstrated asymptomatic subclinical primary infections with one or more EEHV species in many zoo elephants (11–13, 17–19). Characteristic features of these infections involve first a peak of low- to moderate-level viremia detectable in the blood followed, after clearing, by transient shedding in trunk wash secretions for several weeks. EEHV2, EEHV3, EEHV6, and EEHV7 are frequently detected in lung and skin nodules from African elephants and are likely viruses that are endemic to African elephants (9, 10) (V. R. Pearson, personal communication), whereas EEHV1A, EEHV1B, EEHV4, and EEHV5 are likely all natural endemic viruses of Asian elephants (3, 6, 12). Only a single example of a presumed transmission of lethal infection (involving EEHV3A) from an African elephant source to an Asian elephant calf has been observed (1, 10). Satisfactory explanations for the unexpected severity of disease associated with primary infection by these viruses within their natural hosts, where up to 20% of Asian elephant calves appear to be susceptible to severe EEHV1A viremia, as well as any hints about plausible pathological mechanisms, remain to be deciphered.

At present, genomic DNA sequence analysis provides most of the very limited information available about these novel herpesviruses and their unusual virus-host interactions. Partial genotype characterization of pathological DNA samples from more than 60 disease cases worldwide has revealed that the infection patterns are sporadic, not epidemic, with numerous distinct and highly diverged species and strains involved (16). All cases of EEHV1 at different facilities have involved different strains, and although we presume that it can occur, not a single example of spread of any specific EEHV strain from one facility to another has been documented (2, 3, 20, 21). Furthermore, the seven different EEHV1A strains detected in the initial studies in India exhibit nearly all of the same large range of genetic diversity as those seen in North America and Europe, implying that this species on both sides of the diaspora involves a very ancient and diverse population (6). Evidently, the Western zoo strains must all have originated from and were carried within numerous different wild-born elephants imported from Asia.

In the accompanying paper, we describe the intact 206-kb DNA genome sequence of EEHV4B(Baylor) determined recently from a high-quality trunk wash sample collected from a juvenile Asian elephant that survived mild hemorrhagic disease (22). This genome is the first complete example of a member of the second major GC-rich branch of the probosciviruses, which proved to be related to, but very distinct from, the four previously determined intact genomes of EEHV1A, EEHV1B, and EEHV5A from the AT-rich branch of the *Proboscivirus* genus (23–25). Some initial comparative aspects of the overall gene complement and high GC content in the codon wobble positions in coding regions are described in detail there, as well as evidence for both EEHV4A and EEHV4B subtypes. Here, we present a detailed comparison of other contrasting and unique aspects of the genomes of these two major branches, as well as describing a number of additional unusual features that are common to all of the probosciviruses and add to our earlier suggestions that the EEHVs should be designated a novel fourth subfamily of mammalian herpesviruses.

## RESULTS

### Overall structural comparison of the GC-rich and AT-rich branch proboscivirus genomes.

**FIG 1:**
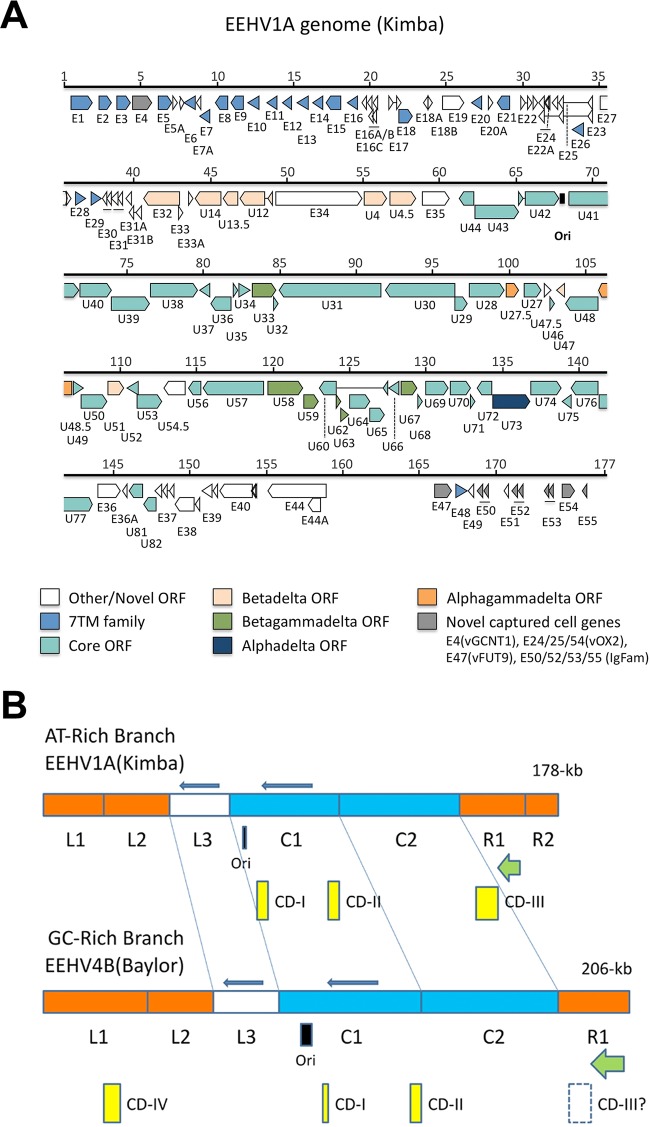
Annotated physical gene map of EEHV1A(Kimba), the prototype example of the AT-rich *Proboscivirus* subgroup and comparison with EEHV4(Baylor). (a) To-scale gene-ORF map of the 178-kb EEHV1A(Kimba) genome based on the data from the work of Ling et al. (23) (GenBank accession no. KC618257) for comparison with the matching map for the newly determined EEHV4B(Baylor) genome (GenBank accession no. KT832477) presented in the accompanying paper (22). Predicted open reading frames (ORFs) are indicated by colored arrows. Gene nomenclature is shown below each of the ORFs. The color key below indicates groups of ORFs shared between all herpesviruses or subsets of subfamilies or multiple paralogues of repetitive genes. Gray arrows indicate novel captured cellular genes, and white arrows indicate novel genes that do not have obvious orthologues outside of the probosciviruses. Thin lines connecting arrows indicate introns. The position of the putative lytic replication origin is marked by a black rectangle. (b) Simplified schematic cartoon comparing the sizes and arrangements of assigned subsegments within the AT-branch EEHV1A(Kimba) and GC-branch EEHV4(Baylor) prototype genomes. The comparative sizes and locations of the CD-I to CD-IV subtype chimeric domains (yellow bars), the predicted Ori-Lyt domain (black bar), and the putative ORF-L plus ORF-K transactivator genes (green arrows) are indicated. Conserved core segments are denoted in blue, and completely novel segments that are unique to the proposed deltaherpesvirus subfamily are shown in orange. In comparison to betaherpesviruses, both segments L3 and C1 are inverted relative to the rest of the genome (indicated by solid blue arrows). Note the complete absence of segment R2 from the EEHV4 version.

Details of the assembly of the 206-kb EEHV4B(Baylor) genome as well as map coordinates and features of the individual genes and proteins are reported in the accompanying paper (22). An annotated physical gene map of the EEHV4(Baylor) genome is also presented in Fig. 1 of the accompanying paper (22), and for comparison, a similar map is presented here in Fig. 1A for an updated version of our previous brief report about the prototype 178-kb EEHV1A(Kimba) genome (23). The chosen genomic orientation for both maps is the same as that used by Richman (26), Ehlers et al. (27), and Ling et al. (23) and represents the best overall match to the genome orientations and alignments in all three other mammalian herpesvirus subfamilies, although the opposite orientation was used by Wilkie et al. (24, 25). Our assignment of and nomenclature for open reading frames (ORFs) within the *Proboscivirus* genus largely follow traditional procedures, with emphasis on identification of protein coding features that match previously identified proteins of other related herpesviruses, and, especially for EEHV4(Baylor), correspond to those used for EEHV1(Kimba) and EEHV5(Vijay). Note that the positions of the EEHV1 genomic terminal repeats as determined by Wilkie et al. (24) mean that for EEHV1A(Kimba) the region between E47 and E55 on the right side in Fig. 1A would be transferred to the left side in the packaged virion DNA. However, that entire region is absent from EEHV4, and for consistency and comparative purposes, we have retained the map coordinates for EEHV1A(Kimba) as used by Ling et al. (23) and Richman et al. (16).

For a convenient gross comparison of the two genomes, the schema in Fig. 1B considers the EEHV1A(Kimba) genome to be divided into seven distinct segments termed L1, L2, L3, C1, C2, R1, and R2. Segment L1 covers genes E1 to E17, L2 covers E18 to E31B, and L3 covers E32 to E35, whereas C1 and C2 together encompass all of the conserved core genes from U27 to U77 (except U81 and U82), and R1 includes E36 to E44, with R2 including E47 to E55. From left to right, segment L1 in EEHV1 (22 kb) has 21 genes mapping between coordinates 1 and 22, but this is expanded to 37 kb (34 genes between coordinates 1 and 37) in EEHV4. Segment L2 in EEHV1 mapping between coordinates 22 and 41 (19 kb, 21 genes) instead occupies positions 37 to 58 (21 kb, 19 genes) in EEHV4. Segment L3 carries 10 genes encompassing 20 kb from coordinates 41 to 61 in EEHV1A and nine genes over 24 kb from coordinates 58 to 82 in EEHV4. Some of the genes in L3 have weak residual homology to parts of an anciently related block with apparent common evolutionary origin within the *Cytomegalovirus* and *Roseolovirus* genera (but it is inverted relative to them in the *Proboscivirus* genus). Core segment C1 (19 genes) extends from coordinates 62 to 102 (40 kb) in EEHV1 compared to coordinates 82 to 131 (49 kb) in EEHV4, and core segment C2 (30 genes) extends from coordinates 102 to 144 (42 kb) in EEHV1 compared to coordinates 131 to 179 (48 kb) in EEHV4. Both C1 and C2 have exactly the same complement of genes in the two branches, of which only two, U47.5 (ORF-J) and U54.5 (ORF-F1), lack homology to any known genes of other herpesviruses. But, unlike in all three genera (*Cytomegalovirus*, *Muromegalovirus*, and *Roseolovirus*) of the traditional betaherpesviruses, the whole of segment C1 (U27 to U44) in the intact genomes of each of the four *Proboscivirus* species and subtypes characterized so far (as well as in EEHV2 and EEHV6) is inverted here relative to segment C2 (U46 to U77). With the exception of core genes U81 and U82 in segment R1 and a couple of captured cellular genes, the other four segments (L1, L2, R1, and R2) all encompass genes that are novel and unique to the *Proboscivirus* genus. Finally, segment R1 (eight to nine genes each) occupies positions 144 to 165 (21 kb) in EEHV1 and positions 179 to 205 (26 kb) in EEHV4, but the entire 12-kb segment R2 (10 to 12 genes, mapping between positions 165 and 177 in EEHV1) is absent in EEHV4.

### Differences in overall gene content, levels of homology, and sizes of orthologous proteins.

Although EEHV4(Baylor) and EEHV1A(Kimba) both have 118 to 119 total genes, there are 25 to 26 genes in both that are not represented in the other. Despite this, the EEHV4 genome is 28 kb larger than EEHV1A, and their overall GC contents differ by 15% (58% versus 43%). As reported in the work of Ling et al. (22), the latter feature is largely caused by the often extremely high GC bias (80 to 99%) within the wobble codon of the majority of the coding regions whether representing core genes or novel genes. In general, the conserved true core proteins (i.e., those shared in common by EEHV4 with all or most other herpesviruses) retain between 65 and 80% amino acid identity over most of their length with their AT-rich branch EEHV counterparts. In contrast, most of the novel *Proboscivirus* genus-specific proteins retain only 30 to 35% amino acid identity over half or less of their length. Indeed, a BLAST search for the best-matching DNA segments of 2 kb or greater in size for EEHV4(Baylor) compared to EEHV1A(Kimba) detected eight loci (totaling 24 kb) that all map within the 82-kb conserved central core segment C in EEHV1A(Kimba) and have nucleotide identities ranging from 63 to 70%. The very best DNA match among core genes was for a 1.1-bp segment at 75% identity within U60 (TERex3), and the best match among the novel parts of the genome was for a 936-bp segment at 71% identity within the E4 (vGCNT1) gene.

One factor contributing to the overall 14.5%-increased size of the EEHV4(Baylor) genome compared to EEHV1A(Kimba) comes from substantially increased sizes for many of the largest novel proteins themselves. For example, there are five likely regulatory proteins and a type-specific glycoprotein that show the most extreme examples of this effect and have increased in size by an average of 70% each. These include the E44 (ORF-L) and E40 (ORF-K) predicted transcriptional lytic cycle trigger proteins, which have increased from 3,900 bp and 2,250 bp up to 6,050 bp and 4,400 bp, respectively, as well as the U42 (MTA) posttranscriptional transactivator, which has increased from 2,300 bp up to 4,070 bp; E36 (ORF-M), which has increased from 1,530 bp up to 2,970 bp; and E37 (ORF-O), which has increased from 1,150 bp up to 2,100 bp. The latter five proteins are also among the least conserved between the two branches, with each displaying just small regions encompassing no more than 15 to 25% of their overall length that have between 42 and 72% amino acid identity.

### Intergenic noncoding domains of EEHV4 are highly enriched in A and T tracts.

In dramatic contrast to the largest coding ORFs, the 21 largest noncoding intergenic domains (totaling 15.1 kb) in EEHV4(Baylor) have GC contents ranging from 31 to 44% and averaging just 35%. Together, this subset of intergenic domains encompasses 323 tracts of successive A or T residues of five or more in length (the largest being a 12-mer). As expected, the intergenic domains are also replete with examples of classic AATAAA poly(A) signal motifs (usually in both directions), but there are a smaller but very striking number of alternating AT nucleotide tracts of between 6 and 16 bp in length in EEHV4(Baylor) that are also most commonly found within the intergenic domains. Overall, there are 76 such alternating AT runs within noncoding regions, plus six in Ori-Lyt and just six others in coding regions (not counting the three largest AT-rich blocks and the chimeric gN-gO-gH-TK gene block, which are all aberrant in this regard).

The high concentrations of A and T homopolymer tracts also apply to most of the noncoding and intergenic domains within EEHV1 and EEHV5, but partly because they are larger and partly because of the overall higher GC content, this is a far more predominant feature of EEHV4. For example, whereas the two adjacent intergenic domains between E19 (ORF-F2) to E20 and E20 to E20B (at map coordinates 40 to 44.9 kb) occupy a total of 2.8 kb and encompass a remarkable 56 A or T runs plus 13 alternating A-plus-T tracts in EEHV4, the same two regions in EEHV1(Kimba) cover just 810 bp with 11 A or T tracts. A full listing of these features within all intergenic and intronic noncoding domains of EEHV4(Baylor) is shown in Table 1. At random, one might expect 200 total examples of 5-mer or longer A and T tracts within a 200-kb genome of average 50% GC content, including just four of 8 nucleotides or longer. However, a global analysis of close to 190 kb of the EEHV4(Baylor) genome (omitting just the three largest AT-rich blocks totaling 10.5 kb and the terminal regions) revealed a total of 855 such motifs altogether, with 169 examples being 8-mers or longer and with all but five of the latter mapping within noncoding regions. Among these, only 187 examples of 5-mer or longer A and T tracts occur within the evaluated coding ORFs, whereas there are a total of 666 such 5-mer or longer A and T tract motifs within the evaluated noncoding regions. Therefore, considering that these noncoding regions (not including the terminal segments and Ori-Lyt) represent about 15% of the entire EEHV4 genome, they are enriched over randomness by at least 32-fold.

**TABLE 1:** Comparison of the locations of A or T tracts in EEHV intergenic domains^a^

EEHV4(Baylor)					EEHV1A(Kimba)				
Locus	Size (bp)	A or T tracts	Alt AT motifs	(A4) adj Init/Term	Locus	Size (bp)	A or T tracts	Alt AT motifs	(A4) adj Init/Term
E1A ups?	260	2	0						
E1A-E1?	360	3	0		E1 ups	350	2	3	
E1-E3	370	4	1	Term	E1-E2	390	3	1	
E3-E3.1	60	1	0		E2-E3	250	2	0	
**E3.1-E3.2**	**580**	**13**	**(1)**						
E3.2-E2A	290	1	(1)	Term					
**E2A-E3.3**	**430**	**11**	**2**						
**E3.3-E3.4**	**920**	**13**	**2**		E3-E4	110	1	1	
E3.4-E4	350	5	0						
E4-E4A	530	2	4	Term	E4-E5	370	4	0	Term
E4A-E4B	70	2	0						
E4B-E4C	200	1	1		E5-E5A	115	1	0	
E4C-E6A	170	5	0		E5A-E6	380	2	3	
E6A-E6B	170	3	0	Init	(E6-E7A)	120	1	0	
**E6B-E6**	**600**	**12 (1)**	(2)	Init	E6-E7alt	270	5	0	Init
E6-E7	280	6	1						
E7-E7B	200	2 (1)	2		E7-E8	320	3	1	
E7B-E9	110	2	0		E8-E9	160	3	0	
E9-E9A	350	3	1	Term	E9-E10	200	1	0	Both
E9A-E9B	40	1	0						
E9B-E9C	60	1	0						
E9C-E10A	Overlap	(1)	0						
E10A-E11	280	3	1	Term	E10-E11	420	0	2	
**E11-E12**	**230**	**8**	**0**		E11-E12	270	5	0	Init
E12-E12A	Overlap				E12-E13	270	4	3	
E12A-E13	420	5	1	Term					
**E13-E14.1**	**440**	**14 (2)**	**2**	Term	E13-E14	240	2	1	
E14.1-E14.2	340	3	0						
**E14.2-E14**	**310**	**9**	**2**	Term					
E14-E15	200	7	2		E14-E15	135	3	0	
E15-E16	230	7	0		E15-E16	360	2	1	
E16-E16D	20	?			**E16-E16A**	**740**	**11**	**3**	
E16D-E17	350	4	0		E16A/B introns	170	(3)	0	
E17-E17A	120	4	0		**E16B-E17**	**860**	**12**	**1**	Init
					E17 intron	110	1	0	
E17A-E18 overlap					E17-E18 overlap				
**E18-E18C**	**420**	**8**	**1**		**E18-E18A**	**1,040**	**15**	**5**	
E18C-E19	580	5	0		E18A-E19	550	7	1	Init
**E19-E20**	**1,100**	**38**	**5**		E19-E20	410	6	1	
**E20-E20B**	**1,700**	**21**	**5**		E20-E20A	430	6	0	
E20B-E20A	20	0	0						
E20A-E21	150	3	0		E20A-E21	230	6	2	
**E21-E22**	**780**	**16**	**0**		E21-E22	720	2	0	
					E22 intron	220	3	0	
E22-E22A	570	6	0	Init	E22-E23	170	2	0	
E22A-E23B	270	4	0		E22A-E23	115	3 (2)	0	
E23B-E24 overlap		0	0		E23-E24	320	4	0	Term
E24B intron	350	2	1		E24 intron	70	1	0	
**E24B-E26**	**420**	**9**	**2**		E24-E26	480	3 (1)	1 (+CA1)	
**E26-E27**	**850**	**10**	**2**		**E26-E27**	**980**	**10**	**0**	
					E27 intron	180	6	1	
E27-E28	60	2	0		E27-E28	230	1	0	Term
E28-E29	230	6	1		**E28-E29**	**320**	**10**	**0**	
E29-E30	130	2	1		E29-E30	40	0	0	
E30-E30A	80	2	0	Term	E30-E31	100	2	1	
E30A-intron	210	3	0		**E31-E31A**	**580**	**10**	**1**	
E30A-E31A	170	3	1		E31 intron	100	1	1	
E31A-E31B overlap									
E31B-E31C	65	0	1						
E31C-E32	75	4 (2)	0		E31A-E32	130	1	1	
**E32-E33A**	**520**	**10**	**2**		E32-E33A	400	7 (3)	0	
E33A-U14	170	6	0	Term	E33A-U14	80	2	0	Init
U14-U13.5	340	5	2		U14-U13.5	90	2	1	Init
U13.5-U12	320	5	1		U13.5-U12	70	2	0	
U12 intron	110	1	0		U12 intron	240	2	0	
U12-E34	480	7	1	Term	**U12-E34**	**340**	**9**	**0**	Term
E34-U4	120	3	0	Term	E34-U4	65	1	0	
**U4-U4.5**	**630**	**16**	**1**		U4-U4.5	50	4	1	Term
**U4.5-E35**	**700**	**16**	**0**		U4.5-E35	360	3	1	
**E35-U44**	**860**	**29**	**0**		**E35-U44**	**640**	**9**	**1**	**Init**
U44-U43 overlap					U44-U43 no gap				
U43-U42	280	4	0	Term	U43-U42	120	3	0	Init
**U42 intron**	**320**	**8**	**9**		U42 intron	145	5	0	
U42-Ori-Lyt (incl)		6	6*		U42-Ori-Lyt (incl)		0	1	
**Ori-Lyt-U41**	**350**	**9**	**0**		Ori-Lyt-U41	550	3	2	
U41-U40	125	6	0	Init	U41-U40	65	2	0	Init
U40-U39 overlap					U40-U39 overlap				Init
U39-U38	160	7	0	Both	U39-U38	25	(1)	0	
U38-U37	210	5	0		U38-U37	50	3	1	Term
U37-U36 no gap					U37-U36 no gap				
U36-U35	110	2	0	Init	U36-U35	140	1	0	
U35-U34	170	1	1	Term	U35-U34	30	0	0	Init
U34-U33	300	6	0		U34-U33	30	3	0	Term
U33-U32 overlap					U33-U32 overlap				
U32-U31	200	3	2	Term	U32-U31	75	3	0	
U31-U30	430	7	1		U31-U30	220	2	2	
U30-U29 no gap					U30-U29 overlap				
U29-U28	250	6	1	Term	U29-U28	50	3	0	
**U28-U27.5**	**140**	**8**	**1**	Term	U28-U27.5	50	0	1	Init
**U27.5-U27**	**600**	**12**	**1**		U27.5-U27	250	3	0	Term
U27-E35A	11	1 (3)	(1)	Term	U27-E35A	80	1	0	
E35A-U45	15	1 (2)	0	Term	E35A-U46 overlap				
U46-U47	85	3	1		U46-U47	25	0	0	
U47-U48 overlap		(2)			U47-U48 overlap				
U48-U48.5 overlap					U48-U48.5 no gap				
U48.5-U49	70	1	0		U48.5-U40 no gap		0	(2)	
U49-U50 overlap					U49-U50 overlap		(4)		Init
U50-U51	85	3	0	Term	U50-U51 no gap (6 bp)		(1)		
U51-U52	190	4	0	Term	U51-U52	120	2	0	Term
U52-U53	95	3	0		U52-U53 overlap		(1)		
U53-U54.5	230	6	0	Term	U53-U54.5	90	3 (1)	1	
U54.5-U56	300	5	1		U54.5-U56	190	2	0	
U56-U57	170	4	0		U56-U57	40	1	0	
U57-U58	545	7	1		U57-U58	165	3	0	Init
U58-U59 overlap					U58-U59 overlap		(3)	0	
U59-U60ex3	230	3	3		U59-U60ex3	35	1 (3)	1	Term
U60ex3-U62	230	3	0		U60ex3-U62	30	2	0	
U62-U63 overlap					U62-U63 overlap		(1)		
U63-U64 overlap					U63-U64 overlap		(1)		
U64-U65 overlap					U64-U65 overlap		(1)		
U65-U66ex2	80	1	0		U65-U66ex2	10	0	0	
U66 intron	210	2	1		U66 intron	180	2	0	
U66ex1-U67	390	6	0		U66ex1-U67	110	3 (2)	(1)	2×Init
U67-U68 no gap					U67-U68 no gap				
**U68-U69**	**510**	**12**	**1**	Init	U68-U69	110	1 (2)	0	
**U69-U70**	**300**	**10**	**2**		U69-U70	60	1 (1)	1	
U70-U71 overlap					U70-U71 overlap				
U71-U72	200	6	1		U71-U72	75	1 (3)	0	
**U72-U73**	**440**	**13**	**0**	Init	U72-U73 no gap				
U73-U74	13	0	0		U73-U74 overlap				Term
U74-U75 overlap		(1)			U74-U75 overlap		(3)?	(1)	
U75-U76 overlap					U75-U76 overlap				
U76-U77 overlap				Term	U76-U77 overlap		(1)		
**U77-E36**	**1,260**	**29**	**0 (1)**	Term	U77-E36	330	2	1	
**E36-U81**	**475**	**14**	**0**		E36-E36A	140	6	0	Term
					E36A-U81	70	3 (1)	0	
U81-U82 overlap					U81-U82 overlap				
U82-E37 overlap					U82-E37 overlap				
E37 intron 2	100	5 (3)	0		E37 intron2	50	1 (1)	1	
E37 intron 1	170	3	1		E37 intron1	75	2	1	
E37-E39A	100	4	(1)		E37-E38 no gap		(1)		
					E38 intron	75	2	1	
E39A intron	80	2	0		E38-E39	130	1	3	
E39A-E40	280	5	1		E39 intron	80	1	0	
					E39-E40	150	0	1 (1)	
**E40-E44**	**960**	**14**	**0**		**E40-E44**	**640**	**12**	**3**	
U44 ups	?				E44 ups dom1	320	6	1	
					**E44 ups dom2**	**650**	**17**	**1**	
					**E44 ups dom3**	**240**	**11**	**1**	

a A or T tracts, A*n* or T*n* homopolymers of 5 nucleotides or more; Alt AT motifs, (AT)*n* of six or more successive alternating A-plus-T nucleotides; numbers in parentheses, additional adjacent copies very close to but not directly within the intergenic domain; Init, initial; Term, terminal; ups, upstream; incl, inclusive; dom, domain; adj, adjacent. Intergenic domains containing eight or more A or T tracts are shown in boldface. *, includes three 10-mers that are part of Ori-Lyt dyad symmetry elements. Details of individual protein (ORF) product sizes and names plus map coordinates for each of the numbered genes listed are presented in Table 1 of the accompanying paper by Ling et al. (22).

Other than the three largest AT-rich blocks of 4.3 kb (vOGT), 4.2 kb (ORF-R), and 1.2 kb (vECTL) in EEHV4(Baylor), which are highly aberrant exceptions and therefore omitted from the analysis above, there are two other very noticeable unusual segments, including the 5.9-kb block encompassing U46 (gO), U47 (gN), gH (U48), U48.5 (TK), U49, and U50, which has no intergenic domains at all (but does include some 20 A or T tracts within coding regions), and the 10-kb block encompassing U73 (OBP), U74, U75, U76 (POR) and U77 (HEL), which also has no intergenic domains (and just one T tract). The 4.2-kb multigene AT-rich block encompassing vOGT also has an unusually high concentration of 18 examples of the 6-mer to 13-mer alternating A-plus-T tracts.

### Detailed comparison of the AT-rich tract features of the intergenic domains in EEHV4(Baylor) compared to EEHV1A(Kimba).

While there are a remarkably large number of these A or T tract features found in both genomes, the extent is greatly enhanced within the GC-rich branch virus EEHV4(Baylor) compared to the AT-rich branch viruses. Table 1 also includes a detailed parallel comparative analysis for EEHV1A(Kimba) of the sizes of all intergenic domains (including introns), together with the total numbers of 5-mer or greater A tracts and T tracts and of 6-mer or greater alternating AT runs that are encompassed within the intergenic domains. The seven largest intergenic domains within EEHV1A(Kimba) all contain eight or more A or T tracts (with the two largest having 12 and 15 of them) and occupy 6.1 kb, but there are 27 such domains in EEHV4(Baylor) with eight or more A or T tracts that together occupy 16.8 kb, with the nine largest having 38, 29, 21, 20, 16, 16, 16, 14, and 14 tracts each. All such intergenic domains containing eight or more tracts are shown in bold in Table 1. Overall, the 103 intergenic noncoding domains found in EEHV4(Baylor) occupy 30.9 kb and encompass a total of 666 A or T tracts plus 70 alternating AT runs, whereas for EEHV1A(Kimba) there are 82 noncoding intergenic domains that occupy 21.9 kb and encompass a total of 291 A or T tracts plus 59 alternating A-plus-T runs. The largest clusters of A or T tracts are distributed about equally across the whole EEHV4 genome with six mapping between E1 and E15, nine mapping between E19 and U44, and nine more mapping between U42 and E44. In contrast, three of the seven largest clusters in EEHV1A(Kimba) lie between E15 and E19, and the 640-bp domain with 12 tracts mapping between E40 (ORF-K) and E44 (ORF-L) is one of the rare examples with a very close match within the EEHV4(Baylor) genome (940 bp, 14 tracts). It is also noticeable that there seems to be a very dramatic trend toward the addition of many more and larger intergenic domains in EEHV4 than in EEHV1 across the entire conserved core-C segment, whereas they are already quite prevalent in the largely novel L1 and L2 segments of both, although the overall concentration of A and T polymeric tracts does still increase greatly in the EEHV4 regions between E1 and E15 and especially between U4 and U44.

### Initiator and terminator codon environment.

In addition to the highly AT-rich nucleotide content of the intergenic domains, there is an extraordinarily high incidence in EEHV4(Baylor) of the presumed initiator ATG codons also being embedded within an adenine-rich nucleotide environment (Table 1). Most commonly, this involves motifs similar to AAAAATG, of which there are 10 perfect matches in EEHV4 as well as 17 that are just 1 bp different and 12 that are 2 bp different, with the substitutions most commonly being T. In comparison, there are just eight initiator codons in EEHV4 that are embedded within the usually quite common CCATGG sequence (plus 15 more that are 1 bp different). Between them, these two types of motifs account for more than half of the expected initiator codons in the virus. Furthermore, there are 25 instances of EEHV4 genes in which the TGA, TAA, or TGA terminator codon lies within the sequence TRRAAAA. In several genes, the terminator is embedded within an A-plus-T block as long as 17 or 18 nucleotides with either none or just one substitution with a G or C nucleotide. Overall, we count 43 genes in EEHV4 with initiators and 63 with terminators that are embedded within highly A-rich or AT-rich sequence environments.

A comparative listing of specific subsets of these motifs within the intergenic domains of the intact EEHV1A(Kimba) genome that have embedded initiator or terminator codons with the sequence AAAAATG or TRRAAAA, respectively, is also presented in Table 1. EEHV1(Kimba) has 8 initiator codons and 14 terminator codons that have exact matches to these motifs, compared to 10 and 25 found in EEHV4(Baylor). However, while the overall effect is about 50% more pronounced in EEHV1A(Kimba), with a total of 61 initiator codons and 75 terminator codons displaying these features, compared to 35 initiators and 58 terminators doing so in EEHV4(Baylor), the phenomenon is nevertheless more obvious and statistically unique within the higher-GC-content EEHV4 genome. In comparison, there are no initiators and just three terminators with the perfect-match version of these motifs in human cytomegalovirus (HCMV), although there are some 13 initiators and 39 terminators out of a total of 164 genes there that would count as being embedded in predominantly AT-rich sequences. Because the AAAAATG-like motifs are poor matches to the standard Kozak rules for high-efficiency mammalian initiator codons, it seems reasonable to predict that this class of herpesviruses might have highly specialized protein synthesis mechanisms for utilizing these unusual initiator and terminator codon environments.

### Multigene families.

**FIG 2:**
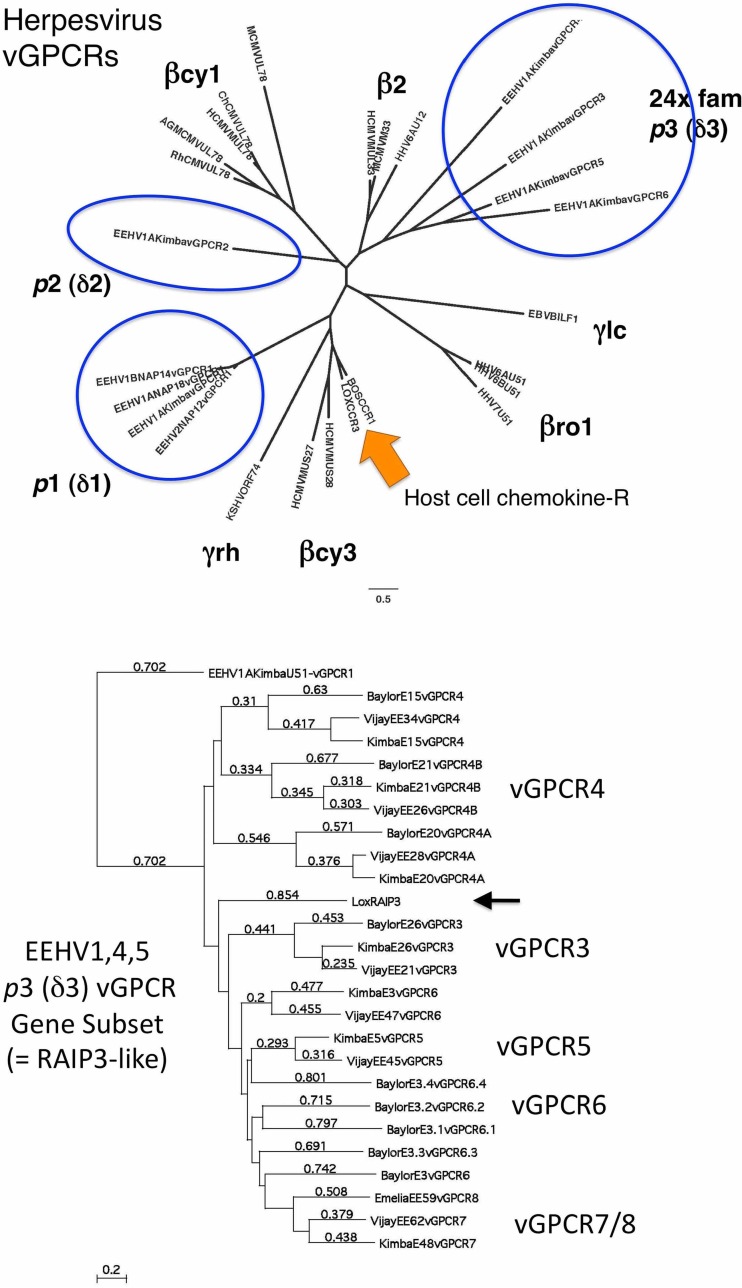
Phylogenetic relationships among the multiple vGPCR-like gene families of the probosciviruses compared to those in the beta and gamma mammalian herpesvirus subfamilies. (Top) Radial Bayesian phylogenetic tree showing the EEHV1 *Proboscivirus* or proposed Deltaherpesvirus versions *p*1 (or δ1) (=vGPCR1), *p*2 (or δ2) (=vGPCR2), and *p*3 (or δ3) (=vGPCR3 to -6) groups of 7xTM-containing vGPCR and chemokine receptor family proteins compared to key human representatives of all genera within the mammalian beta- and gammaherpesvirus subfamilies, including the *Lymphocryptovirus* (=γlc) (BILF1), *Rhadinovirus* (=γrh) (ORF74), *Roseolovirus* βro1 (U51) and β2 (U12), or *Cytomegalovirus* βcy1 (UL78), β2 (UL33), and βcy3 (US27, US28) versions. The three distinct branches of the EEHV1 vGPCR-like proteins are shown encompassed by either blue ovals (*p*1 and *p*2 or δ1 and δ2) or a blue circle (*p*3 or δ3). The arrow denotes two mammalian host cell chemokine receptors, CCR1 (Bov) and CCR3 (Lox), that are likely to exemplify or resemble the original source of the captured viral orthologues. Note that the cytomegalovirus US27 and US28 chemokine receptors (βcy3) are the most similar to the mammalian host versions, implying that they may have been the most recently acquired, whereas the other herpesvirus groups were very anciently acquired. (Bottom) Linear distance-based Bayesian phylogenetic tree comparisons of the vGPCR-related subset of the *p*3 or δ3 multigene family from the EEHV4(Baylor) GC-rich branch *Proboscivirus* genome compared to their counterparts in EEHV1A(Kimba) (vGPCR3 to -7), EEHV1B(Emelia) (vGPCR8 only), and EEHV5A(Vijay) (vGPCR3 to -6). The positions of the prototype vGPCR3 to -8 versions from EEHV1 in the tree are indicated. Note that for this tree the highly diverged but related EEHV1(Kimba) U51 (vGPCR1) protein is used as the outgroup, whereas the closest-matching host cell orphan vGPCR member (retinoic acid-inducible protein 3 [RAIP3], arrowed) branches between the two major superclusters of vGPCR4, 4A and 4B, compared to vGPCR3 and vGPCR5 through to vGPCR8. Bar and individual branch number values denote relative genetic divergence.

One of the most dramatic distinguishing features of the probosciviruses compared to all other mammalian herpesvirus groups is the very large number of often tandemly repeated members of the 7xTM gene family. Overall, we recognize 24 members of the 7xTM protein family in EEHV1, 25 in EEHV5, and 28 in EEHV4. Excluding the two chemokine-R-like proteins U51 (vGPCR1, *p*1, or δ1) and U12 (vGPCR2, *p*2, or δ2), which clearly had different independent origins (see Fig. 2, top), the 26 remaining mostly smaller 7xTM domain family genes within the third EEHV (*p*3 or δ3) set presumably arose through multiple tandem-duplication events after the original capture of an ancestral version (or versions), although E1, for example, now has a large extended N-terminal Ser-plus-Thr-rich domain. Overall, they represent the largest paralogous gene family known in any herpesvirus genome and apparently also one of the oldest in terms of levels of divergence. Some of the presumed original tandem-repeat structure still manifests as a series of between five and eight adjacent genes all arranged in the same orientation (e.g., E1 to E6 and E11 to E16 in the EEHV1 genome map in Fig. 1B as well as in the EEHV4 gene map in Fig. 1 of the work of Ling et al. [22]), but other segments have apparently subsequently undergone either inversions or interruptive dispersal by insertion of other unrelated genes. These events evidently occurred so long ago that many paralogues across the EEHV1 and EEHV4 *p*3 (or δ3) 7xTM gene family have diverged too far to retain measurable protein homology with one another, but several sets of subgroupings do remain, and clear residual relationships occur between most of the EEHV1 and EEHV4 orthologues, with much closer relationships still between the EEHV1 and EEHV5 versions (see below).

**FIG 3:**
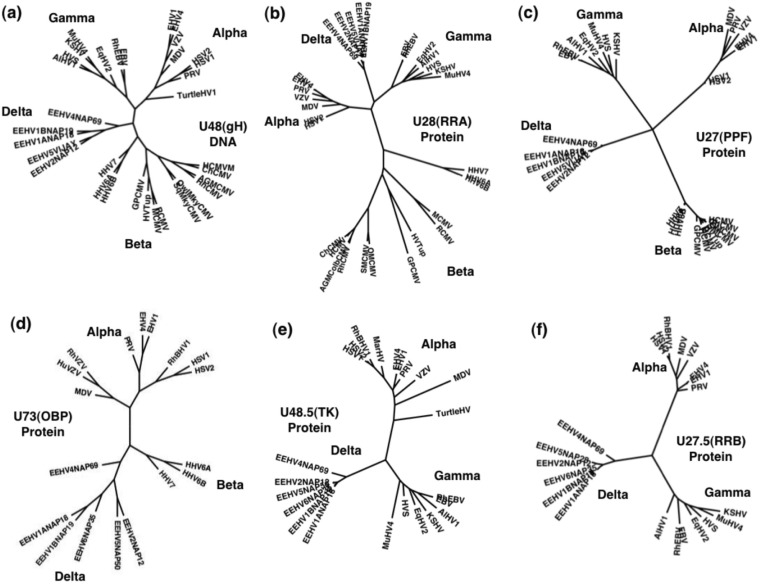
Unique cladal divergence patterns of selected *Proboscivirus* core and alpha-gamma-*Proboscivirus* class proteins from their orthologous counterparts in other mammalian herpesvirus subfamilies. Radial distance-based Bayesian phylogenetic trees were generated in MEGA5 from MUSCLE alignments for the following six intact proteins of EEHV4(Baylor), including U48 (gH) (a), U28 (RRA) (b), U27 (PPF) (c), U73 (OBP) (d), U48.5 (TK) (e), and U27.5 (RRB) (f), representing examples of true core (a, b, and c) or alpha-gamma (αβ) (d and f) or just alpha-beta2 (αβ2) (e) class genes compared to representative orthologues of these proteins from the other three mammalian herpesvirus subfamilies. α, alphaherpesviruses; β, betaherpesviruses; γ, gammaherpesviruses; δ, probosciviruses or proposed deltaherpesviruses.

In the accompanying paper by Ling et al. (22), all 26 members of the *p*3 (or δ3) 7xTM domain protein family of EEHV4 were grouped into five related multigene subsets, with the assignments being based on a combination of PBLAST domain-match identity values with the prototype genes E3, E6, E14, E15, and E18, as well as on branching pattern criteria derived from the phylogenetic tree alignment information shown there in Fig. 3 for the full set of intact proteins. The last two subgroups within the *p*3 (or δ3) set 7xTM gene family consisting of E15 (vGPCR4), E20, and E21, plus E18 and E28, are conserved in all three EEHV lineages, but the numbers of family members in the other three subgroups vary. In addition, E2, E8, and E10 of EEHV1A(Kimba) have either been deleted or diverged too far to be recognizable in EEHV4, and three new members, E2A, E7B, and E10A, have seemingly been acquired or retained only in this lineage. Comparative examples of and mechanisms for the expansion and contraction of tandemly repeated vCXCL and vGPCR gene clusters in Old World primate cytomegaloviruses have been described and discussed previously (28).

Remarkably, despite there being at least 13 different members of the membrane-anchored vIg (E50-like) family that are found (albeit in different numbers and arrangements) within the EEHV1A, EEHV1B, and EEHV5 genomes, there is not a single gene of this type present in EEHV4. Several of the vIgFam proteins show significant homology to mammalian host CD48, and they all cluster together with vOX2-1 and vFUT9 at one end of the genome, but because this whole 12-kb gene block (segment R2) is missing from the EEHV4 genome, it represents a major differentiating feature of the GC-rich versus AT-rich branch probosciviruses.

There are two other sets of duplicated genes (U54.5 and E17) present within all three EEHV lineages. The former, located within the core segment C2, occupies a position equivalent to the characteristic HCMV UL83-84-85 DURP gene family of phosphorylated tegument proteins. However, because this protein lacks any residual amino acid identity to the cytomegalovirus, muromegalovirus, or roseolovirus versions (although it does have a similar multiple beta-barreled sheet structure), it was designated ORF-F1 in EEHV1(Kimba) (23). Interestingly, there is also a second highly diverged copy of this gene in all three *Proboscivirus* lineages, designated E19 or U54.5-2 (ORF-F2), which has 25% amino acid identity to the ORF-F1 versions over 77% of its length but is instead located among the large 7xTM gene family block within the novel L2 segment.

### Captured cellular genes.

In addition to the standard core herpesvirus enzymes and DNA replication proteins, such as uracil DNA glycosylase (UDG), RRA, RRB, thymidine kinase (TK), and polymerase (POL), which all have measurable homology to equivalent presumed ancestral host proteins and likely represent ancient gene capture events, there are several more novel proteins in EEHV4(Baylor) that have significant homology to host cell proteins and that were evidently much more recently acquired (Table 2). These include first the E4 (vGCNT1) acetylglucosamine transferase, which is the most conserved noncore protein of all between EEHV1 and EEHV4 (61% identity over 68% of its length). This captured host cell enzyme, which is unspliced and therefore likely to have been acquired originally as a cDNA form, also retains 61% identity over 68% of its length to the host *Loxodonta africana* (Lox) version. The mammalian host cell version plays an important role in control of extravasation in leukocytes by its ability to generate O-glycan-linked sugar modifications of mucins. EEHV4 also encodes a second type of captured O-linked or UDP-acetylglucosamine transferase, E9A (vOGT), also known as B36NT3, HRNT1, or O-GLNAC, as well as a captured C-type lectin, E16D (vECTL), which are both unspliced, but neither is present in either the EEHV1 or EEHV5 genome. vOGT has 53% identity to the host African elephant enzyme, and vECTL has 42% identity over 28% of its length to an *L. africana* C-type lectin. Note that the two major types of rat cytomegalovirus (RCMV), RCMV-M(Maastricht) and RCMV-E(English), each have either one or two captured versions of C-type lectins also (vRCTL or vRCTL2), but those versions all retain four or five exons (29–31).

**TABLE 2:** List of recently captured host cell genes present in *Proboscivirus* genomes^i^

Gene/protein name	Size (aa)	Present in EEHV type(s)	% identity to orthologue^a^		Normal functional role in host	Other virus(es) in which found, % identity
			*Loxodonta*	Human		
vGCNT1 (E4)	536	All EEHVs^b^	68 (58)	58 (68)	Protein glycosyl	BoHV4 (γ), 52 (68)
vFUT9 (E47)	387	EEHV1A, -1B, -5	46 (91)	41 (97)	Protein glycosyl	Mimiviruses
vOGT1 (E9A)	414	EEHV4A, -4B	53 (74)	50 (74)	Protein glycosyl	Nil
vECTL (E16D)	185	EEHV4A, -4B	48 (56)	39 (80)	NK cell receptor	MuHV8 (β), 31 (95); SMCMV (β); CrHV (γ); TorHV (α); ChHV(Scuta); cowpox and fowlpox viruses
vOX2-1^c^ (E54)	295	EEHV1A, -1B, -5	83 (65)	67 (81)	Cell-cell ligand, Ig-fam, two Ig domains, TM anchor (T-cell, MC-Mph)	EqHV5 (γ) E11, 74 (68); MuHV8 (β), 65 (72); KSHV (γ) U14, 42 (61); HHV6 (β) U85; EBV (γ); frog HV1; Yaba poxvirus
vOX2-2^d^ (E25)	260	EEHV1A, -1B, -5	28 (68)	30 (64)	Same as above, two Ig domain, TM anchor	EEHV1 E54, 32 (69); KSHV E14, 26 (88); HHV6 U85, 32 (38)
vOX2-3^e^ (E24)	176	EEHV1A, -1B, -5	40 (39)	42 (46)	Same as above, one Ig domain	EqHV5 E11, 39 (39); acidic C-term
vOX2-4^f^ (E23)	264	EEHV1A, -1B	30 (70)	32 (69)	Same as above, two Ig domain, acidic C-term	EEHV1 E54, 32 (69); EqHV5 E11, 20 (70)
vOX2-V^g^ (EE22A)	260	EEHV5	34 (70)	34 (68)	Same as above, two Ig domain, TM anchor	EEHV1 E54, 36 (71); EqHV5 E11, 36 (64); MuHV8 e127, 29 (75)
vOX2-B^h^ (E24B)	132	EEHV4A, -4B	41 (65)	40 (71)	Same as above, one Ig domain	EEHV1 E54, 38 (67); EqHV5 E11, 42 (65); EEHV5 EE22A, 38 (71)
vGPCR1 (δ1) (U51)	405	EEHV4	17 (51) (Mu)		Serotonin R; opioid R; CCR1; MCMV, swinepox	EqHV2 E1 17 (59); HCMV US28 16 (47); FeHV1 (α); HHV6
vGPCR1 (δ1) (U51)	376	EEHV1A, -1B, -5	20 (57) (Hu)		Atyp Chem R4 (T-cell, MC-Mph); CCR11, Angio RII	EqHV2 E1 28 (28); sheep, goat, fowlpox
vGPCR2 (δ2) (E12)	782	EEHV4	18 (38)		Angiotensin RII, opioid R, CXCR1 somatostatin R	
vGPCR2 (δ2) (E12)	608	EEHV1A, -1B, -5	23 (51) (Hu)		CCR2; CCR1, -4, -5, -6, -9	MCMV M33, 21 (59); RCMV, HCMV, EqHV2, HTup
vRAIP3 (δ3) (E3)	315	All EEHVs (incl EEHV4)	22 (80) (Hu)	Retinoic acid-induced protein 3	Multiple paralogues in 7xTM family, includes EEHV1 vGPCR3 to vGPCR8	
vCXCL1 (E36A)	105	EEHV1A, -2, -5	Missing third Cys		Chemokine ligand (inhibitory?)	Some cytomegaloviruses, roseoloviruses; some rhadinoviruses
vCD48 (EE44A)	222	EEHV5 (-1A, -1B) (vIgFam)	32 (84)		BLAST1, T-cell regulation	Some strains of EEHV1; SMCMV, OMCMV; EqHV2 (γ), poxviruses
ORF-C (E34)	1,898	EEHV1A, -1B, -5	33 (10)		FAM186 repeats	

a Amino acid identity (fractional length of protein with homology), both as percentages.

b All EEHVs means EEHV1A, EEHV1B, EEHV4A, EEHV4B, EEHV5A, and EEHV6 at least.

c Unspliced.

d Exons 1, 2, and 3 of EEHV1.

e Exons 4a, 4b, and 5 of EEHV1.

f Exons 1, 2, and 5 of EEHV1.

g Exons 1, 2, 3, and 4 of EEHV5.

h Exons 1 and 2.

i Abbreviations: incl, including; BLAST1, B-lymphocyte activation marker; Bo, bovine; Eq, equine; Hu, human; Mu, murine; MC-Mph, monocyte-macrophage; TM, transmembrane; Ig, immunoglobulin; term, terminal; MCMV, murine cytomegalovirus; SMCMV, squirrel monkey cytomegalovirus; CrHV, cricetid herpesvirus; TorHV, tortoise herpesvirus; ChHV, chimpanzee herpesvirus; FeHV1, feline herpesvirus 1; Angio, angiotensin; OMCMV, owl monkey cytomegalovirus; Atyp Chem, atypical chemokine; Htup, *Herpesvirus tupaia*.

Most strikingly, EEHV4 has evidently lost (or never acquired) the E47 (vFUT9) fucosyl transferase and the highly conserved E54 (vOX2-1) protein (98% match to *L. africana* over a 200-amino-acid [aa] block encompassing both the immunoglobulin v and c domains), which map together with the CD48-like membrane-anchored vIg domain family genes within the highly variable E47 to E55 gene block in segment R2 that is a signature novel feature of both the EEHV1 and EEHV5 genomes. One of the roles of FUT9 in mammalian host cells is terminal additions of fucose to produce Lewis antigens, and the OX2 (CD200) ligands act in moderating interactions with other myeloid cells expressing CD200 receptors for dampening excessive monocyte-macrophage-mediated responses to infection by viral and bacterial pathogens.

Another characteristic feature of the EEHV1A, EEHV1B, and EEHV5 genomes is the presence of a second cluster of tandemly repeated and much more diverged (and thus likely more anciently captured) OX2 (CD200)-like proteins with only about 30% residual amino acid homology to the host version, which have the unusual feature of retaining a greatly shortened predicted spliced intron from between exon 3 and exon 4 of the mammalian OX2 gene. These were designated E25 (vOX2-2) and E24 (vOX2-3) with a possible alternatively spliced E23 (vOX2-4) version in EEHV1A(Kimba) also. The second of these proteins (vOX2-3) has exchanged the typical C-terminal membrane anchor domain present in both vOX2-1 and vOX2-2 and the host OX2 versions with an acidic tail, indicating a role as a likely soluble secreted version. In EEHV5(Vijay), the acidic-tailed version is not present, but instead, there is a third tandemly duplicated (and membrane-anchored) copy. In contrast, the situation is different again within EEHV4, in which just a single captured vOX2-like protein (vOX2-B or E24B) has been retained. This version is only 130 aa in length and, like the third version in EEHV5, contains just a single rather than two immunoglobulin domains. The vOX2-B protein displays just 37% identity over 76% of its length (80 aa) to E54 (vOX2-1) of EEHV1A(Kimba), as well as very similar levels of homology to a gammaherpesvirus version encoded by equine herpesvirus 5 (EqHV5) (E11), and 36% identity over 79% to host *L. africana* OX2 but has seemingly acquired a new upstream short first exon (16 aa). Although the smaller single Ig-domain protein vOX2-B of EEHV4 most likely had a common evolutionary capture origin with one or more of the vOX2-2 or vOX2-3 orthologues present at the same location in EEHV1 and EEHV5, it shows measurable identity only to the host OX2 version and to E54 (vOX2-1) of EEHV1, but not to the other vOX2s, so it not possible to determine to which of the other EEHV paralogues it is most closely related.

Finally, all the EEHV genomes contain several kinds of vGPCR-like genes with seven transmembrane domains (7xTM family), with both U51 (vGPCR1, *p*1, or δ1) and U12 (vGPCR2, *p*2, or δ2) but not the others having features resembling those of host chemokine receptors. Although they map at the same relative genomic positions as their presumed orthologues UL78 and UL33 in cytomegaloviruses and roseoloviruses, both have nevertheless diverged to such an extent and in such a manner that they only occasionally register in BLASTP searches as having residual homology with just one or a few of the betaherpesvirus orthologues but have equal or better matches to both host and poxvirus versions. The unspliced EEHV4 version of U51 (vGPCR1) is 42% identical over 93% of its length to the EEHV1A(Kimba) version and otherwise most closely resembles the host Lox CCR1, CCR2, CCR3, and CXCR4 (all with domains having 25% identity over 130 to 199 aa). Similarly, the predicted-to-be-spliced EEHV4 U12 (vGPCR2) is 50% identical over 53% of its length to the EEHV1A(Kimba) version, but otherwise it matches best to the host Lox CCR1, CCR6, and CCR9 proteins (domains with 20% identity over 295 aa), which is more than that to the spliced betaherpesvirus positional orthologues. The EEHV4 versions of U12 and U51 also match at about the same level to a subset of cellular alpha- or beta-chemokine receptors.

U51 (vGPCR1) and U12 (vGPCR2) are significantly larger than any of the numerous remaining novel tandemly repeated *p*3 (or δ3) 7xTM domain family paralogues in all EEHV genomes, with vGPCR2 especially having a large extended C-terminal Ser-plus-Thr-rich domain. Clearly, both had distinct and separate evolutionary capture histories, probably occurring as very ancient events shared within a common ancestor of the *Cytomegalovirus*, *Roseolovirus*, and *Proboscivirus* genera before they themselves diverged. As mentioned above, we have designated the EEHV U51 (vGPCR1) and U12 (vGPCR2) proteins as *p*1 and *p*2 (or δ1 and δ2) set herpesvirus vGPCRs, whereas those from the third large novel subgroup (vGPCR3 to -8) are referred to as the *p*3 (or δ3) set for comparison with the many other captured cellular vGPCR gene lineages present within the other mammalian herpesvirus subfamilies. A radial protein level phylogenetic tree is presented in Fig. 2 (top) that shows the evolutionary relationships among the EEHV1 prototypes of proboscivirus *p*1 (δ1), *p*2 (δ2), and several *p*3 (δ3) vGPCRs (circled) together with selected herpesvirus vGPCRs representing the sets that we have designated here βcy1 (US27, U28-like), βcy2 (UL33), and βcy3 (UL78) from the cytomegaloviruses (human cytomegalovirus [HCMV]); βro1 (U51) and βro2 (U12) of roseoloviruses (human herpesvirus 6 [HHV6]); γlc (Epstein-Barr virus [EBV]) of the lymphocryptoviruses; and γrh (Kaposi’s sarcoma-associated herpesvirus [KSHV]) of the rhadinoviruses, as well as the prototype cellular versions BovCCR1 and LoxCCR3.

Among the remaining very large family of smaller *p*3 (or δ3) 7xTM domain proteins encoded by EEHVs, we originally considered five in EEHV1A(Kimba) to qualify by BLASTP homology searches (23) as being related to the cellular orphan G-protein-coupled receptor (GPCR) C-5-A/C/D group, including having about 27% protein identity (over 45 to 80% of their length) to RAIP3 (also known as RAIG1 or PEIG1). Consequently, in the original EEHV1A(Kimba) genome annotation, E26, E15, E5, E3, and E48 were designated vGPCR3, vGPCR4, vGPCR5, vGPCR6, and vGPCR7, respectively, by this criterion, and E20 (vGPCR4A) and E21 (vGPCR4B), which display just a slightly lower level of homology to RAIP3, have now also been added. We also designated another highly diverged member of this family found only in EEHV1B(Emelia) as vGPCR8 (EE62). The latter matches the vGPCR7 versions found at similar positions in EEHV1A(Kimba) (E48) and EEHV1A(Raman) (EE62), although by only 40% at the protein identity level.

A linear distance-based protein phylogenetic tree showing the relationships among all members of this third vGPCR subgroup (*p*3 or δ3) in EEHV1A(Kimba), EEHV5A(Vijay), and EEHV4B(Baylor) is presented in Fig. 2 (bottom). These data show that EEHV5 also encodes versions of all but one of these same vGPCR family proteins that average 40 to 60% divergence. However, for EEHV4, whereas E48 (vGPCR7) and EE59 (vGPCR8) are both absent, clearly identifiable versions of the vGPCR3, vGPCR4, vGPCR4A, and vGPCR4B proteins are retained (each at about a 30% to 40% identity level over 50 to 90% of the length), but the situation with regard to vGPCR5 and vGPCR6 is more complicated. We interpret that there are five ancient tandemly duplicated paralogues of either vGPCR5 or vGPCR6 or both present in EEHV4. These all have about equal matches (28 to 38% identity over 40 to 70% of the protein) to both vGPCR5 and vGPCR6, but they map on the left side of the vGCNT1 gene, whereas in EEHV1 and EEHV5, E3 (vGPCR6) maps to the left and E5 (vGPCR5) maps to the right of E4 (vGCNT1). Therefore, we have given E3.1, E3.2, E3.3, and E3.4 the designations vGPCR6.1, vGPCR6.2, vGPCR6.3, and vGPCR6.4, respectively. Most likely, all four of the latter may have been deleted from an ancestral genome within the AT-rich branch, whereas E5 (vGPCR5) was deleted from the GC-rich branch.

### The EEHV versions of several herpesvirus core proteins display unique cladal branches relative to all other mammalian herpesvirus versions in phylogenetic trees.

We previously presented a series of radial phylogenetic trees at both the DNA and protein levels (16) showing the unique branching positions of the EEHV1A and other AT-rich branch proboscivirus versions for 12 examples of both highly conserved and less well conserved proteins relative to orthologues from each of the three currently designated mammalian herpesvirus subfamilies, including representatives of all or a broad range of other defined genera as well as still-unassigned members. In Fig. 3, we show one example of a similar DNA distance-based radial phylogenetic tree for a representative true core gene, U48 (gH) (Fig. 3a), as well as similar protein level trees for two more core genes, U28 (RRA) and U27 (PPF) (Fig. 3b and c), all with the new GC-rich EEHV4(Baylor) versions added. Two alpha-gamma-delta (αγδ) class genes, U48.5 (TK) and U27.5 (RRB) (Fig. 3e and f), as well as the alpha-beta2-delta (αβ2δ) class gene U73 (OBP) (Fig. 3d), are also shown. Because of the absence of these TKs, RRBs, and origin-binding proteins (OBPs) from all cytomegaloviruses and almost all betaherpesviruses, the proboscivirus versions form a new novel and highly distinctive third mammalian branch compared to the alpha- and gammaherpesvirus versions. The tree for U74 (PPF), the polymerase processivity factor (Fig. 3c), is especially intriguing, showing a dramatic “four-leaf clover” pattern. Similarly, the EEHV versions of RRA (Fig. 3b) are hugely diverged from those of the *Cytomegalovirus*, *Muromegalovirus*, and *Roseolovirus* genus versions from within the betaherpesvirus subfamily and instead branch together with the alpha- and gammaherpesvirus versions.

**FIG 4:**
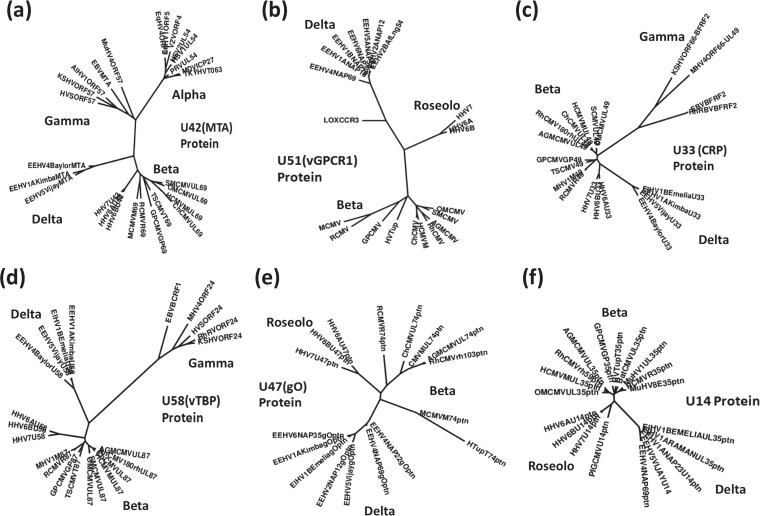
Unique cladal divergence patterns of the *Proboscivirus* MTA posttranscriptional transactivator and other selected proteins from their orthologous counterparts in other mammalian herpesvirus subfamilies. Radial distance-based Bayesian phylogenetic trees were generated in MEGA5 after alignments in MUSCLE for the following intact proteins of EEHV4(Baylor) and all other available EEHV species: U42 (MTA), the conserved posttranscriptional regulatory transactivator (a); U51 (vGPCR1) (b); U33 (CRP) (c); U58 (vTBP) (d); U47 (gO) (e); and U14 (f), representing examples of the shared beta-gamma-*Proboscivirus* (β*p* or βδ) and beta-*Proboscivirus* (β*p* or βδ) classes of genes. All are compared to representative orthologues from the other three mammalian herpesvirus subfamilies. α, alphaherpesviruses; β, betaherpesviruses; γ, gammaherpesviruses; δ, probosciviruses or proposed deltaherpesviruses.

All mammalian herpesviruses encode in common an immediate-early class of posttranscriptional regulatory proteins known as ICP27 (alpha), MTA (gamma), or UL69 (beta), which fall into one of three very distinctive groups in phylogenetic trees that are characteristic for each of the three acknowledged subfamilies. The EEHV-encoded orthologues of this protein, U42 (MTA-E), again form a related but fourth separate cladal group (Fig. 4a) that is further diverged from the nearest members of the other three subfamily groups than the entire genetic distance displayed across all members within either the alpha-, beta-, or gammaherpesvirus subfamilies. The radial protein trees for two of the six βδ (or β*p*) class core genes, U33 (CRP) and U58 (vTBP), are also included as examples in Fig. 4c and d to illustrate that the proboscivirus versions form a very distinctive third branch that is separate from both the betaherpesvirus and gammaherpesvirus versions. Even for the small number of EEHV genes described above that have apparent common ancestry shared uniquely with the betaherpesviruses, the proboscivirus versions as exemplified by U51 (vGPCR1), U47 (gO), and U14 in Fig. 4b, e, and f, respectively, are still far further diverged from all the betaherpesvirus versions than are the *Roseolovirus* versions from the highly clustered *Cytomegalovirus*, *Muromegalovirus*, and other unclassified CMV-like versions. These results all further illustrate and amplify the points made previously about the novel evolutionary nature of most genes within the *Proboscivirus* genus compared with the betaherpesvirus subfamily (16).

### Novel set of proboscivirus-specific likely transcriptional transactivator proteins.

Each of the three currently recognized mammalian herpesvirus subfamilies has a pair of dedicated nuclear transcriptional regulatory proteins (often referred to as the major immediate-early proteins) that map adjacent to one another and have unique characteristic features that define them as being alpha-, beta-, or gammaherpesvirus types irrespective of the genus to which they have been assigned. For alphaherpesviruses, the ICP4 and ICP0-like proteins perform these roles, whereas in betaherpesviruses it is the MIE1 and MIE2-like proteins and in gammaherpesviruses the equivalents of RTA and ZTA. In each case, there is no resemblance between these proteins across subfamily barriers, whereas all orthologous members across the multiple genera within a subfamily either have residual detectable protein homology or have easily recognizable similar structural features. Although multiple different mechanisms are involved, each of these six proteins (in the well-characterized human versions especially) function as transcriptional transactivators of many downstream viral genes and act either singly or together as the primary “lytic cycle triggers.” They often also have immediate-early class expression characteristics and are mostly spliced with highly hydrophilic characteristics, including Pro-rich and Ser-rich blocks and both highly acidic and highly basic domains, as well as having one or more classic nuclear localization signal (NLS) motifs present. Four of them are direct DNA-binding factors (ICP4, MIE2, RTA, and ZTA) that can also have secondary roles in initiating DNA replication.

If the EEHVs truly represent betaherpesviruses as they were originally designated, we would certainly expect them to encode proteins mapping somewhere to the right of U81 (UDG) and U82 (gL) in the genome that have residual protein identity to the MIE1 and MIE2 proteins of HCMV, HHV6, and the other betaherpesvirus versions. However, despite the fact that there are five large proteins mapping within the anticipated location, three of them (ORF-O, -P, and -Q) are novel envelope glycoproteins, but none of the others have homology to either MIE1 or MIE2 or any other known herpesvirus proteins. Nevertheless, assuming that the probosciviruses would indeed likely encode one or more similar dedicated nuclear transcriptional transactivators, the most plausible candidates for this role are those two remaining novel hydrophilic proteins, namely, E44 (ORF-L) and E40 (ORF-K), which map in a leftward orientation close to the right end of all EEHV genomes (Fig. 1A and B). In EEHV1A, ORF-L is 1,307 aa in size, whereas the EEHV5 version is 1,182 aa and the EEHV4 version is 2,018 aa. While all three versions of ORF-L have by far the more dramatic nuclear transcriptional transactivator-like features, including Pro-rich and Ser-rich stretches, as well as localized highly basic and highly acidic features and two plausible NLSs (16), the second immediately adjacent protein would also have to be considered partly because its juxtaposed downstream position could allow them both to be coordinately regulated. E40 (ORF-K) does encode another large hydrophilic protein that could also be a nuclear protein and that comprises 741 aa in EEHV1A, 684 aa in EEHV5A, and 1,465 aa in EEHV4. Most significantly, neither of them nor any other EEHV protein has detectable primary amino acid sequence identity with any of those other three pairs of functional dedicated immediate-early mammalian herpesvirus regulatory proteins from the alpha, beta, or gamma subfamilies. Note that we are not considering here the several other herpesvirus proteins that do qualify as unusual and special unique or minor immediate-early class transactivators (such as VP16 of herpes simplex virus [HSV]; TRS1, vICA, and vMIA of HCMV; or Meq in the avian alphaherpesvirus Marek’s disease virus [MDV]) but just the conserved dedicated pairs of coregulated primary transcriptional regulatory proteins of each of the three mammalian herpesvirus subfamilies.

**FIG 5:**
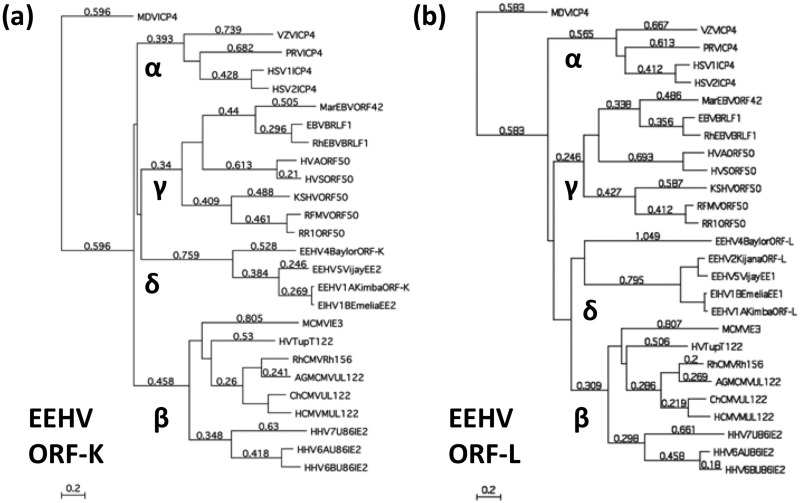
Phylogenetic divergence of the predicted *Proboscivirus* transcriptional transactivator proteins (ORF-L and ORF-K) from those of all three of the currently designated mammalian herpesvirus subfamilies. (a) Comparative linear protein-level Bayesian phylogenetic tree after alignments in MUSCLE for four versions of E40 (ORF-K) from both the AT-rich and GC-rich branches of the *Proboscivirus* genus relative to potential key representative orthologues from the alphaherpesvirus (α) (ICP4), betaherpesvirus (β) (MIE2), and gammaherpesvirus (γ) (RTA) subfamilies. Note that the potential *Proboscivirus* (δ) (ORF-K) versions display no significant amino acid identity relationships with any of the other three subfamilies of mammalian herpesvirus regulatory transactivator proteins. Bar and individual branch number values denote relative genetic divergence. (b) Comparative linear protein-level Bayesian phylogenetic tree after alignments in MUSCLE for five versions of E44 (ORF-L) from both the AT-rich and GC-rich branches of the *Proboscivirus* genus relative to potential key representative orthologues from the alphaherpesvirus (α) (ICP4), betaherpesvirus (β) (MIE2), and gammaherpesvirus (γ) (RTA) subfamilies. Note that the potential *Proboscivirus* (δ) (ORF-L) versions display no significant amino acid identity relationships with any of the other three subfamilies of mammalian herpesvirus regulatory transactivator proteins. Bar and individual branch number values denote relative genetic divergence.

Although both the alpha ICP0-like and beta MIE1-like proteins have each diverged too far to display residual amino acid homology across all the different genera within those two subfamilies, the four other primary transactivators do all still display measurable homology across the whole extent of the range of variants within each subfamily. We shall also ignore the ZTA (and Meq) proteins here because they are clearly a distinctive case, having been acquired as captured bZip family members from the host, and there are no similar proteins found in the EEHV genomes. Interestingly, among the remaining three dedicated transcriptional transactivator families, whereas all versions of the ICP4-like proteins and the MIE2-like proteins retain conserved subdomains just within their C-terminal segments, the RTA-like proteins instead have conserved subdomains that map at their N termini. In comparison, both the ORF-L and ORF-K proteins are more like the former with conserved subdomains just within the C-terminal segments. Although there may be no genetic or evolutionary relationships at all between the three primary mammalian herpesvirus subfamily groups of dedicated transcriptional regulatory proteins, the relative divergences among individual members within the ICP4, MIE2, and RTA protein families can all be illustrated on the combined distance-based phylogenetic trees shown in Fig. 5a and b. When the EEHV1A, EEHV1B, EEHV5, and EEHV4 versions of the ORF-K and ORF-L proteins are also included, the results emphasize the complete absence of any significant alignment relationships between any of the three major known transcriptional transactivator family groups themselves, or between any of them with either of the two candidate putative *Proboscivirus* equivalents. On the other hand, the ORF-K and ORF-L versions from both the AT-rich and GC-rich *Proboscivirus* branches each fall into distinct clades, as also do all members within the alpha (ICP4), beta (MIE2), and gamma (RTA) subfamilies themselves. Note especially in the current context that the MIE2 proteins of the *Cytomegalovirus*, *Muromegalovirus*, and unassigned bat, guinea pig, and tree shrew CMV-like viruses within the betaherpesvirus subfamily all still show a pronounced close relationship even with the *Roseolovirus* versions but not at all with either of the candidate *Proboscivirus* ORF-K or ORF-L proteins. Furthermore, the AT-rich and GC-rich branch versions of ORF-L (Fig. 5b) are further diverged from each other than are any two groups within the alphaherpesvirus or betaherpesvirus subfamilies and about equal to that of the *Rhadinovirus* and *Lymphocryptovirus* branches of the gammaherpesviruses. However, the two branches of ORF-K (Fig. 5a) are diverged from one another to about the same level as are the Old and New World primate versions of RTA, as well as similar to that between HSV and varicella-zoster virus (VZV) or the tree shrew and human versions of CMV. Overall, these results, like most of the other phylogenetic trees shown above and previously, indicate that the evolutionary relationship between the *Proboscivirus* genus and all of the currently defined betaherpesvirus genera is far more diverged than between any other pair of genera within the alpha-, beta-, or gammaherpesvirus subfamilies.

## DISCUSSION

The present study expands the overall description presented in the accompanying paper (22) of the many novel features found within the intact 206-kb genome of EEHV4, the first example of a GC-rich branch *Proboscivirus*. The emphasis here is on the extraordinary distribution of AT-rich intergenic domains between the well-defined GC-rich coding regions, plus other novel unique and generic features about the genomes, especially the captured cellular genes and large 7xTM domain protein gene families of all known EEHV types.

One of the issues that was covered extensively in the accompanying paper is the high wobble codon GC content bias found in almost all of the well-conserved EEHV4 genes, as well as in many of the novel *Proboscivirus*-specific genes as well. We noted there that the phenomenon was almost completely absent within the AT-rich branch EEHV genomes, as well as within a small subset of EEHV4 genes that either occur within several small aberrant AT-rich locations or appear to be recently captured or rapidly evolving genes specific to the GC-rich branch. Considering that the high GC bias seemed to correlate well with older well-established viral genes and perhaps also late-lytic-class viral genes, it was particularly intriguing to find that the captured E4 (vGCNT1) gene in EEHV4 (which maps in the left-side novel area and is unique to the probosciviruses) provided one of the most extreme examples of high-GC wobble bias. For more insights in that regard, we were prompted to address and compare the question of high-GC wobble codon bias in all of the apparently recently captured cellular genes of these viruses. The analysis revealed first that even the EEHV1 version of vGCNT1 displays a remarkably high GC content (52%) and wobble position bias (68%) for that virus (although nowhere near the values of 63% and 96.6% for the EEHV4 version, respectively). Even the Lox host version of GCNT1 shows a little bit of the same trend, with 45% overall GC content and 54% at the wobble position. Similarly, the related and evidently very recently captured vGCNT3 gene present in the gammaherpesvirus bovine herpesvirus 4 (BoHV4), which is 90% and 96% identical to the host bovine GCNT3 isoform version at the DNA and protein level, respectively, has an overall GC content of 51% and wobble codon GC content of 65% (whereas the intact viral genome is 41% GC content). Note that none of the captured vFUT9, vOX2-1, nor vOX2-2/3 genes of EEHV1 show any wobble content GC bias, nor do the vECTL and vOGT genes of EEHV4, but they all have much lower overall GC content as well. We also indicated in the other paper that the U41 (major DNA-binding protein [MDBP]) and U57 (major capsid protein [MCP]) genes of EEHV4 represented typical examples of high wobble codon GC bias. In comparison, the orthologous MDBP and MCP genes in both HCMV and EBV, two other herpesviruses of similar overall 58% to 59% GC content as EEHV4, do trend toward wobble codon GC bias with values of 72% to 79%, although still significantly below the 89% and 86% levels shown by the EEHV4 versions. Overall, these observations tend to support the notion that the captured vGCNT1 genes of probosciviruses were acquired as very ancient events, presumably more than 35 million years ago, when the AT-rich and GC-rich branches are estimated to have diverged (9).

There is an ongoing unresolved issue about whether the *Proboscivirus* genus members should be classified as outliers in the betaherpesvirus subfamily or instead as a distinct new subfamily of mammalian herpesviruses designated the deltaherpesviruses (9, 16, 23, 32). In comparison to the total of 161 genes carried by the 242-kb HCMV (33), all three lineages of Asian EEHV genomes now sequenced each retain just 50 of these same genes overall but lack the other 111 HCMV genes, including the betaherpesvirus and *Cytomegalovirus* genus-specific blocks between RL01 and RL13 (7 genes); UL01 to UL31 (28 genes); UL36 to UL43 (7 genes); UL72, UL83, UL84, and UL116 to UL150 (32 genes); US1 to US34 (31 genes); and IRS1/TRS1. The genes retained in common with HCMV include the 44 true core genes and the six beta-gamma (βγ)-specific genes, which include gene blocks from UL44 to UL57 (15 genes) plus UL69 to UL71 (3 genes) and UL73 to UL105 (29 genes) plus UL114 and UL115 (2 genes), as well as just UL33 to UL35 and perhaps UL27 (5 genes) from the left-side betaherpesvirus-specific noncore gene block, and finally UL74 (gO) and UL78 (vGPCR1) from the core region. Similarly, with regard to overall gene organization compared to the prototype *Roseolovirus* HHV6 (159 kb), just like EEHV1 and EEHV5, EEHV4 has the same large inversion of the 40-kb core gene blocks I, II, and III from U27 to U44 (segment C1), as well as a second inversion involving the 24-kb segment L3, including U4 and the U12 to U14-like genes, but the EEHVs all lack the DR, U1 to U3, and U5 to U11 genes as well as all of the region from U15 to U26 (including vICA, vMIA, and the US22 family), and also everything from U83 to U100 (at least 35 genes overall).

Both the assigned and as-yet-unassigned members (such as tree shrew and guinea pig herpesviruses and two groups of bat herpesviruses) of the betaherpesvirus subfamily also all have large differences in gene content, as also do human and great ape CMVs, for example, compared to Old World and New World primate CMVs, but the EEHVs lack many more genes still from among those previously regarded as key characteristic genes of this subfamily. Most especially, all *Cytomegalovirus* and *Roseolovirus* members and unassigned betaherpesviruses have a related set of immediate-early-like dedicated nuclear transcriptional regulatory proteins (known as MIE1 and MIE2) that are completely different from the similarly well characterized functionally equivalent regulatory proteins of the alphaherpesvirus (ICP4 and ICP0) or gammaherpesvirus (RTA and ZTA) subfamily. But, it turns out that the probosciviruses do not encode any proteins related to these. Assuming that the EEHVs do indeed have functionally equivalent dedicated nuclear transcriptional transactivators, the two most plausible expected candidates (designated ORF-K and ORF-L) would represent a fourth completely different set of putative transcriptional regulatory proteins among known mammalian herpesviruses.

Phylogenetically, as described in detail previously (16), almost all of the common core proteins in EEHVs branch separately from and occupy an intermediate position between the betaherpesvirus and gammaherpesvirus versions, without being particularly closely aligned with either group. As demonstrated here in Fig. 3 and 4, the EEHV versions of several other typical herpesvirus genes (RRA, PAF, TK, and OBP especially), including the posttranscriptional regulator U42 (MTA-E), as well as others that are present only in subsets of mammalian herpesviruses, also all form novel distinctive cladal branches in phylogenetic trees.

Interestingly, the AT-rich and GC-rich branches of the probosciviruses have distinct but overlapping sets of relatively recently captured or pirated host cellular genes (Table 2). While both branches encode the vGCNT1 acetylglucosamine transferase, only the GC-rich branch has the second vOGT type of O-linked acetylglucosamine transferase and only the AT-rich branch encodes the vFUT9 fucosyl transferase. A related version of acetylglucosamine transferase is also encoded within just one other known herpesvirus, bovine herpesvirus 4 (BoHV4), a member of the gammaherpesvirus subfamily, but that protein (Bo17, vGCNT3) is actually a GCNT3 isoform with very-high-level DNA and protein identity (96%) to the host bovine GCNT3 protein, implying a much more recent acquisition event. The captured vECTL gene present in EEHV4 (but not found in either EEHV1 or EEHV5) also matches best with one of the two captured vRCTL proteins (34) encoded by RCMV (murine herpesvirus 8 [MuHV8]), as well as less well with several other beta- or gammaherpesvirus versions. In contrast, although a version of FUT9 is also encoded by several mimiviruses, we are not aware of any other virus type that has captured an OGT-like gene.

On the other hand, the probosciviruses represent the sixth lineage of herpesviruses known to have captured a version of the host cell OX2 or CD200 protein. However, the AT-rich branch EEHVs have acquired this on at least two occasions, with one relatively “new” unspliced version (vOX2-1) in both EEHV1 and EEHV5 retaining an amazing 98% amino acid identity with the host version across a 200-aa domain encompassing both its central constant and variable domains (exon 3 and exon 4). Despite this extraordinarily high level of protein conservation, the “new” vOX2-1 genes of EEHV1, EEHV2, and EEHV5 all display 20% nucleotide divergence from each other and from the host gene (J.-C. Zong, S. Y. Heaggans, S. Y. Long, E. M. Latimer, and G. S. Hayward, unpublished data), indicating that the original gene capture event must nevertheless have occurred up to 20 million years ago before they diverged from one another. In contrast, the other “old” captured version (vOX2-2, -3, and -4) is both highly diverged and still predicted to be spliced and evidently underwent an ancient duplication event in EEHV1 and was triplicated in EEHV5. However, only one very different and shortened version of the older spliced vOX2 type is retained in EEHV4 (vOX2-B). Interestingly, BLAST-P searches with these EEHV vOX2 protein sequences led to the identification of the BARF1 (or CSL1) protein of human EBV with 24% (64%) amino acid identity to EEHV1 E54 (vOX2-1) as another previously unrecognized and highly diverged captured version of cellular OX2 (CD200). None of the EEHV genes encoding the pirated vGCNT1, vOGT, or vFUT9 enzymes nor the vOX2-1 or vECTL protein show any predicted splicing features.

Although there are no formal criteria for classifying herpesviruses into subfamilies, the large distinctions between and novel features of the biology of the EEHVs compared to betaherpesviruses and all other mammalian herpesviruses, as well as the major differences in genomic organization, including both orientation and gene content, but most especially the relative positions of individual proteins in phylogenetic trees, all make a strong case for separate subfamily status for the probosciviruses. Overall, we consider the absence of either any protein homology or any specific structural resemblances of ORF-K and ORF-L, the best candidates for putative *Proboscivirus* transcriptional regulators, to those of any of the three currently recognized mammalian subfamilies, including the betaherpesvirus *Roseolovirus* branch, to be the most compelling argument of all to consider designating them members of a new fourth deltaherpesvirus subfamily, rather than lumping them together with the already hugely diverged betaherpesviruses. Considering the unique early divergence of the Afrotheria group (including their elephantid hosts and the mastodons) from the Eutheria that gave rise to all other later evolving groups in the radiation of placental mammals, it is hardly surprising that a cladal group of herpesviruses that evidently coevolved together with them starting more than 100 million years ago are themselves highly diverged from the other alpha, beta, and gamma subfamilies of mammalian herpesviruses.

## MATERIALS AND METHODS

### DNA sequence analysis and comparisons.

Clustal alignments and Bayesian distance based-phylogenetic trees were generated in MEGA5 based on MUSCLE alignments or in MacVector 12 as described previously (16).
